# Comparative Metabolic Analysis of Different *Indica* Rice Varieties Associated with Seed Storability

**DOI:** 10.3390/metabo15010019

**Published:** 2025-01-05

**Authors:** Fangxi Wu, Yidong Wei, Yongsheng Zhu, Xi Luo, Wei He, Yingheng Wang, Qiuhua Cai, Huaan Xie, Guosheng Xie, Jianfu Zhang

**Affiliations:** 1 Rice Research Institute, Fujian Academy of Agricultural Sciences, Fuzhou 350019, China; wufangxi@faas.cn (F.W.); weiyidong@faas.cn (Y.W.); zhuyongsheng@faas.cn (Y.Z.); luoxi@faas.cn (X.L.); hewei@faas.cn (W.H.); wangyingheng@faas.cn (Y.W.); caiqiuhua@faas.cn (Q.C.); huaanxie@faas.cn (H.X.); 2 Ministry of Agriculture and Rural Affairs of the People’s Republic of China, Key Laboratory of Crop Ecophysiology and Farming System in the Middle Reaches of the Yangtze River, College of Plant Science and Technology, Huazhong Agricultural University, Wuhan 430070, China

**Keywords:** rice (*Oryza sativa* L.), seed storability, metabolome, metabolism pathway

## Abstract

Seed storability is a crucial agronomic trait and indispensable for the safe storage of rice seeds and grains. Nevertheless, the metabolite mechanisms governing *Indica* rice seed storability under natural conditions are still poorly understood. Methods: Therefore, the seed storage tolerance of global rice core germplasms stored for two years under natural aging conditions were identified, and two extreme groups with different seed storabilities from the *Indica* rice group were analyzed using the UPLC-MS/MS metabolomic strategy. Results: Our results proved that the different rice core accessions showed significant variability in storage tolerance, and the metabolite analysis of the two *Indica* rice pools exhibited different levels of storability. A total of 103 differentially accumulated metabolites (DAMs) between the two pools were obtained, of which 38 were up-regulated and 65 were down-regulated, respectively. Further analysis disclosed that the aging-resistant rice accessions had higher accumulation levels of flavonoids, terpenoids, phenolic acids, organic acids, lignans, and coumarins while exhibiting lower levels of lipids and alkaloids compared to the storage-sensitive rice accessions. The Kyoto Encyclopedia of Genes and Genomes (KEGG) pathway enrichment analysis indicated that several biosynthesis pathways were involved in the observed metabolite differences, including alpha-linolenic acid metabolism, butanoate metabolism, and propanoate metabolism. Notably, inhibition of the linolenic acid metabolic pathway could enhance seed storability. Additionally, increased accumulations of organic acids, such as succinic acid, D-malic acid, and methylmalonic acid, in the butanoate and propanoate metabolisms were identified as a beneficial factor for seed storage. Conclusions: These new findings will deepen our understanding of the underlying mechanisms governing rice storability.

## 1. Introduction

Rice (*Oryza sativa* L.) is a vital staple crop for more than half of the world’s population due to its crucial role in global food security. However, the aging process during seed storage results in an annual loss of approximately 3% of rice yields [1]. On the other hand, the deterioration of seed germination ability due to short seed storability poses a major challenge for high yields and better quality [2]. Therefore, enhancing seed storability is of paramount importance in order to guarantee the safety and preservation of rice grains and seeds during natural storage conditions.

Seed storability refers to the longevity of seeds after storage. It is regarded as a significant agronomic trait for maintaining seed fitness post-harvest [3]. Generally, seed storability is influenced by genetic and environmental factors throughout the plant growth, seed maturation, and post-harvest stages. Improving the seed storage environment demands substantial resources and labor costs, which is not economically feasible. Nevertheless, leveraging genetic factors through breeding programs has proven to be highly effective in enhancing the storability of rice seeds and grains. Over the past decade, more than 70 quantitative trait loci (QTLs) associated with seed storability have been identified using QTL location analysis and association mapping approaches under natural or artificial aging conditions [4]. However, only a few QTLs, such as *qSS-9* or *qGP-9*, *qSS1*, and *qSS3.1*, have been precisely mapped [5,6,7]. To date, several genes associated with storage resistance have been successfully cloned. For example, the cloned genes include the lipid-peroxidation-related genes *OsLOX1*, *OsLOX2*, *OsLOX3*, and *OsLOX10* [1,8,9,10], the *OsALDH7*, *OsGLYI3*, and *OsAKR1* genes responsible for scavenging toxic substances [11,12,13], the reactive oxygen species-scavenging genes *OsHSP18.2* and *OsMSRB5* [14,15], the protein repair genes *OsPIMT1* and *PIMT2* [16,17], the antioxidant accumulation gene *Rc* [18], and the antioxidant enzyme genes *OsCSD2* and *OsCSD2* [19]. These identified genes have significantly enhanced our understanding of the underlying mechanisms of storage resistance to a certain extent. However, due to the complexity of these genetic mechanisms, QTLs and genes associated with seed storage mechanisms have not been well elucidated.

Metabolomics is primarily used to examine a wide range of small-molecule metabolic intermediates such as lipids, nucleic acids, amino acids, peptides, carbohydrates, organic acids, ketones, aldehydes, amines, steroids, vitamin signaling molecules, hormones, and polyphenols [20,21,22]. Metabolomics can be classified into targeted metabolomics, non-targeted metabolomics, and a novel integrated method known as widely targeted metabolomics [23]. The widely targeted metabolomics technique offers greater precision than non-targeted analysis [23,24,25,26].

The composition and content of accumulated metabolites in newly matured rice seeds have often been determined, which directly dictate the subsequent storage capacity under external environmental conditions. Therefore, in this study, 375 rice core accessions from 47 countries were collected and evaluated for their seed storability based on the variation among four groups (*Basmati*, *Indica*, *Aus*, *Japonica*). Then, by employing the Indica rice group with its large variation as a representative, a resistant storage pool of five extremely resistant varieties and a sensitive storage pool of five highly sensitive varieties were selected and subjected to UPLC-MS/MS widely targeted metabolomics analysis to evaluate the metabolic disparities among the rice seeds with varying storabilities. Consequently, a number of differential accumulated metabolites (DAMs) and significant metabolic pathways related to storage stability were identified to elucidate the underlying new mechanisms governing rice storability in the rice cultivars.

## 2. Materials and Methods

### 2.1. Plant Materials

A rice population consisting of 375 cultivated rice accessions was collected from the 3K Rice Genome (3K-RG) and obtained from the Institute of Crop Sciences, Chinese Academy of Agricultural Sciences in Beijing, China. These accessions were selected as the core collections from 47 countries, representing major rice-growing regions worldwide (Table S1). These varieties were cultivated in the Fujian Nanfan base, located in Tengqiao Town, Haitang District, Sanya City, Hainan Province.

### 2.2. Natural Seed Aging Treatment

The newly harvested rice seeds were stored at room temperature in the Rice Research Institute, Fujian Academy of Agricultural Sciences. Fifty healthy and filled seeds were randomly chosen for treatment after 3 months and 24 months, with three replicates for each sample.

### 2.3. Seed Germination Test

The treated seeds were sown on two layers of filter paper [4] and germinated in an incubator set at a temperature of 30 °C with a relative humidity of 75% under a light cycle of 14 h per day for a duration of 14 days. The germination percentage was calculated by dividing the number of germinated seeds by the total number of seeds.

### 2.4. Sample Preparation and Extraction for Metabolomic Analysis

We selected five *Indica* rice varieties with extremely resistant storage and extremely sensitive storage, respectively, to form an aging-resistant *Indica* rice pool (AR) and an aging-sensitive *Indica* rice pool (AS). Each variety was represented by 50 shelled seeds. The samples were frozen using a vacuum freeze-drier (Scientz-100F, Ningbo, China), followed by grinding with a mixer mill (MM400, Retsch, Haan, Germany) and zirconia beads at 30 Hz for 1.5 min until they turned into powder. Lyophilized powder (50 mg) was dissolved in 1.2 mL of a 70% methanol solution, vortexed every 30 min for 30 sec (6 × replicates), and refrigerated overnight at 4 °C to ensure proper dissolution. Subsequently, the samples were centrifuged at 12,000 rpm for 3 min and filtered before UPLC-MS/MS analysis [20]. To assess the repeatability of the measurement process, quality control (QC) samples were prepared by combining all sample extracts, with one QC sample analyzed after every three regular samples.

### 2.5. Chromatographic Mass Spectrometry Acquisition Conditions

The sample extracts were analyzed using UPLC (Ultra Performance Liquid Chromatography, ExionLC™ AD, Shanghai, China, https://sciex.com.cn/ (accessed on 27 December 2024) coupled with MS/MS (tandem mass spectrometry). The liquid-phase conditions included a chromatographic column (Agilent SB-C18, 1.8 μm, 2.1 mm × 100 mm, Santa Clara, CA, USA), and a mobile phase consisting of solvent A (pure water with 0.1% formic acid) and solvent B (acetonitrile with 0.1% formic acid). The elution gradient started at 95% A and 5% B for the first 9 min, followed by a linear gradient to reach 5% A and 95% B within that time frame. This composition was maintained for an additional minute before adjusting back to the initial conditions of 95% A and 5% B within another minute, which was then kept constant for the next four minutes. The flow rate was set at a velocity of 0.35 mL per minute, while the column oven temperature was maintained at a constant value of 40 °C throughout the analysis period. An injection volume of precisely two microliters was used in each run.

The effluent from the UPLC system was directed towards an ESI-triple quadrupole linear ion trap (QTRAP)-MS instrument (Framingham, MA, USA) for further analysis. The ESI source operation parameters were as follows: the source temperature was set to be stable at 550 °C; the ion spray voltage (IS) was applied in the positive polarity mode and had a value of 5500 V, whereas the negative polarity mode had a value of −4500 V; GSI (ion source gas I), GSII (ion source gas II), and CUR (curtain gas) were adjusted to 50 psi, 60 psi, and 25 psi, respectively; CAD (the collision-activated dissociation) was set to high. QQQ scans were acquired in the MRM mode with nitrogen as the collision gas set to medium. DP (declustering potential) and CE (collision energy) for individual MRM (multiple reaction monitoring) transitions were optimized accordingly [23,27]. A specific set of MRM transitions corresponding to metabolites eluted during each specific period was monitored.

### 2.6. Multivariate Statistical Analysis

Multivariate statistical analysis was conducted to investigate the accumulation of accession-specific metabolites, employing hierarchical clustering analysis (HCA), Pearson’s correlation coefficient (PCC), principal component analysis (PCA), and orthogonal partial least squares discriminant analysis (OPLS-DA) on the metabolic data of each sample. Heatmaps with dendrograms were utilized to visually represent the HCA results for both metabolites and samples, while the cor function in R was employed to calculate PCC values for the samples. The heatmap package in R was used for generating heatmaps based on both PCC and HCA outcomes. PCA was performed using the GraphPad Prism v9.01 software (GraphPad Software Inc., La Jolla, CA, USA) to display the original-state metabolomic data and variables. Prior to the HCA and PCA analyses, metabolite data were scaled by unit variance. To compare metabolic characteristics among the different rice varieties, an OPLS-DA model was constructed using the MetaboAnalystR 3.2 package in the R software. Log_2_ transformation of the metabolite data followed by mean centering was applied before conducting OPLS-DA analysis to improve normality assumptions. A permutation test with 200 permutations was executed to prevent overfitting issues. DAMs screening criteria included a variable importance in projection (VIP) ≥ 1 in the OPLS-DA model, an absolute Log_2_FC (fold change) ≥ 1, and a *p*-value < 0.05. Venn diagrams were employed to illustrate the number of DAMs.

### 2.7. KEGG Annotation and Enrichment Analysis

The different metabolites were annotated using the KEGG compound database (https://www.kegg.jp/kegg/compound/ accessed on 27 December 2024) and mapped to the KEGG pathway database (https://www.kegg.jp/kegg/pathway.html accessed on 27 December 2024) for annotation and enrichment analysis. Metabolite sets enrichment analysis (MSEA) was performed on pathways with significantly regulated metabolites, and their significance was assessed using *p*-values from hypergeometric tests.

## 3. Results

### 3.1. The Difference in Seed Storability of Global Rice Core Accessions After Natural Aging for Three Months and Two Years

**Figure 1:**
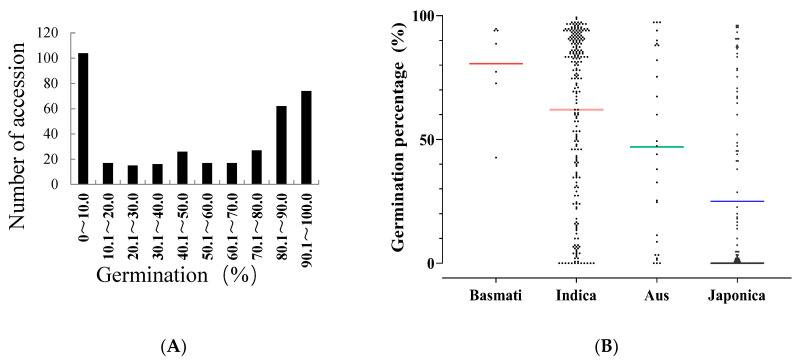
Disparity in the seed storability of the whole population of 375 rice core accessions and four groups from 47 different countries. (**A**) Distribution of and variations in seed germination percentages in 375 accessions after natural aging treatment for 24 months. (**B**) Scatter dot plot illustrating the seed germination percentages in the four rice groups (*Basmati*, *Indica*, *Aus*, and *Japonica*) with different colored means.

In this study, we collected a total of 375 rice core collections from 47 different countries (Table S1), which were categorized into four rice groups (*Basmati*, *Indica*, *Aus*, and *Japonica*) [27]. The seed germination was evaluated after natural aging for 3 months and 24 months in the Fuzhou laboratory, Fujian Province, China, to assess the variation in seed storability among the global rice core accessions. Following the 3 months of natural aging, the average germination rate was recorded as 96.87%, with values ranging from 91.33% to 100% (Table S2). Significant variations in the seed germination percentage were observed within the population after natural aging for 24 months, with values ranging from 0% to 99.33% and an average of 50.57% (Table S2, Figure 1A). The distribution curve of the seed germination percentages among the collection of rice core accessions showed a continuity form, indicating substantial differences in storability among these core accessions. Moreover, there was a notable disparity in seed storability among these four groups (Figure 1B), with *Basmati* exhibiting superior storability, followed by the *Indica*, *Aus*, and *Japonica* groups, respectively.

### 3.2. Metabolic Profiling Analysis in AR and AS Pools

To further dissect the disparity in and metabolite mechanisms of seed storability in the *Indica* group popularized in Fujian, China, five *Indica* rice varieties exhibiting extreme resistance to storage aging and five varieties exhibiting extreme sensitivity to storage aging were selected, forming a pool of *Indica* varieties with high resistance to aging (AR) and a pool of *Indica* varieties with high sensitivity to aging (AS), respectively. As a result, a significant disparity was observed between the five aging-resistant *Indica* varieties and the five aging-sensitive *Indica* varieties after undergoing natural aging for 24 months. The seed germination percentages of the AR group exceeded 94%, while the AS group exhibited almost negligible germination capability (Table 1).

**Figure 2:**
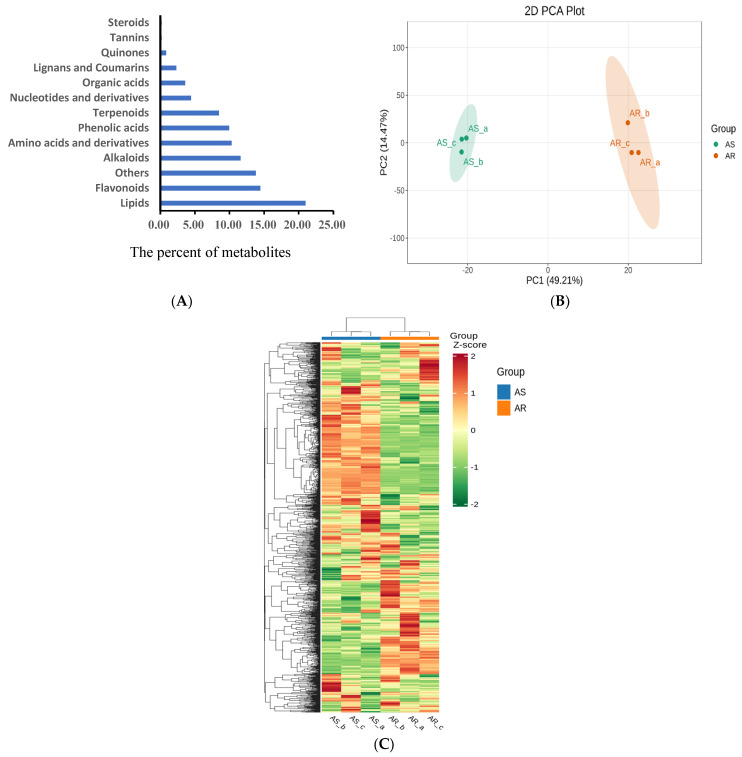
Analysis of metabolite profiles in AR and AS pools. (**A**) Classification of 1098 identified metabolites. (**B**) Scatter plot from the PCA model representing different rice storage pools. The abscissa PC1 and ordinate PC2 represent scores of the first and second principal components, respectively. (**C**) Overall clustering heatmaps of all differentially accumulated metabolites from the two pools. Each scatter represents a sample, with the color and shape indicating different groups.

Then, to further elucidate the mechanisms of storage tolerance, comprehensive metabolic profiling of the AR and AS groups was analyzed by using a widely targeted metabolite analysis based on the UPLC-MS/MS method. The UPLC-MS/MS method enabled us to accurately and precisely identify a total of 1098 metabolites, encompassing lipids (20.98%), flavonoids (14.44%), alkaloids (11.59%), amino acids and derivatives (10.30%), phenolic acids (9.94%), terpenoids (8.46%), nucleotides and derivatives (4.42%), organic acids (3.59%), lignans and coumarins (2.30%), quinones (0.83%), tannins (0.18%), as well as others compounds (13.80%) (Figure 2A, Table S3).

The unsupervised PCA model disclosed that two principal components accounted for 63.68% of the total variance, with PC1 contributing to 49.21% and PC2 to 14.47%, respectively. This indicated a significant difference in the metabolic profiles between the AR and AS groups (Figure 2B). HCA was carried out based on the relative differences in the metabolites contents. Three biological replicates demonstrated the repeatability and reliability of the data. The HCA figure further confirmed the significant differences in the metabolic profiles observed through the PCA analysis (Figure 2C).

### 3.3. Identification of Differently Accumulated Metabolites in AR and AS Pools

**Figure 3:**
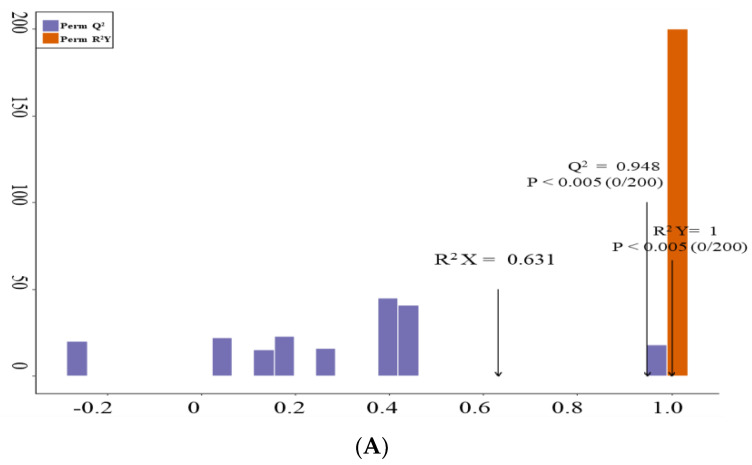

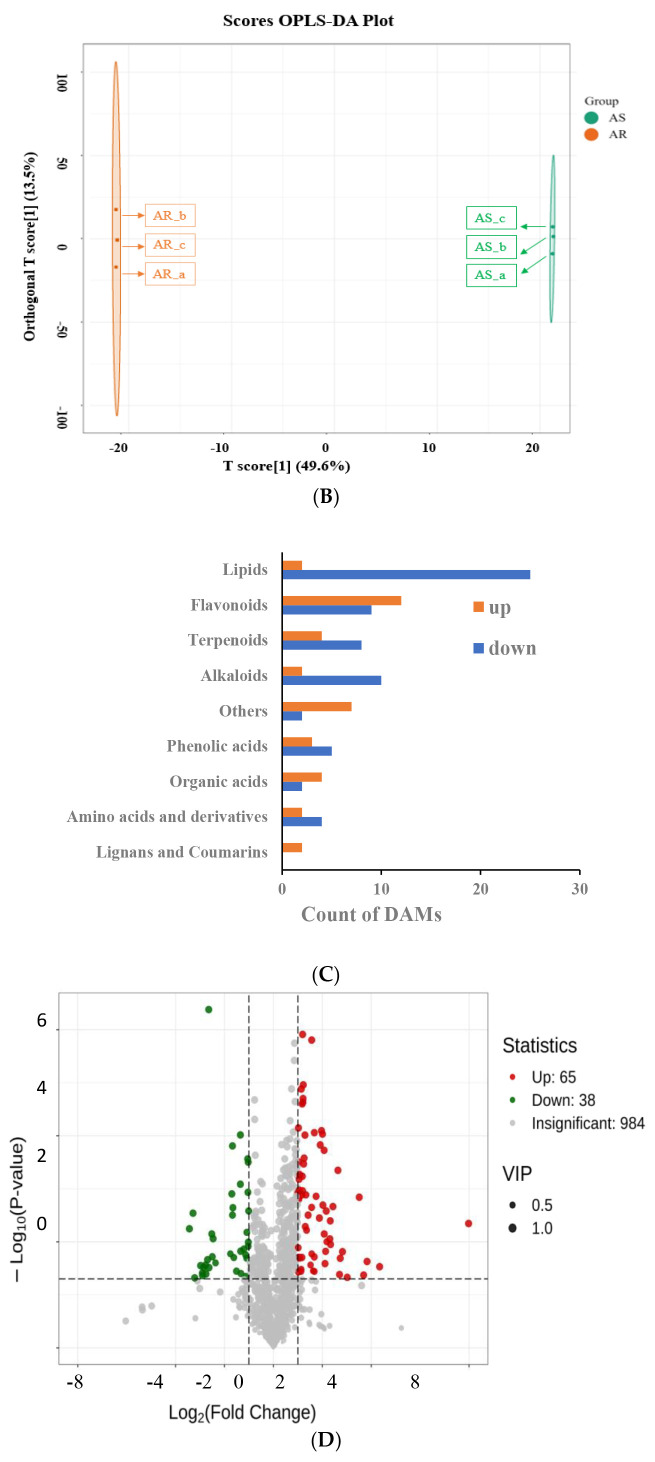

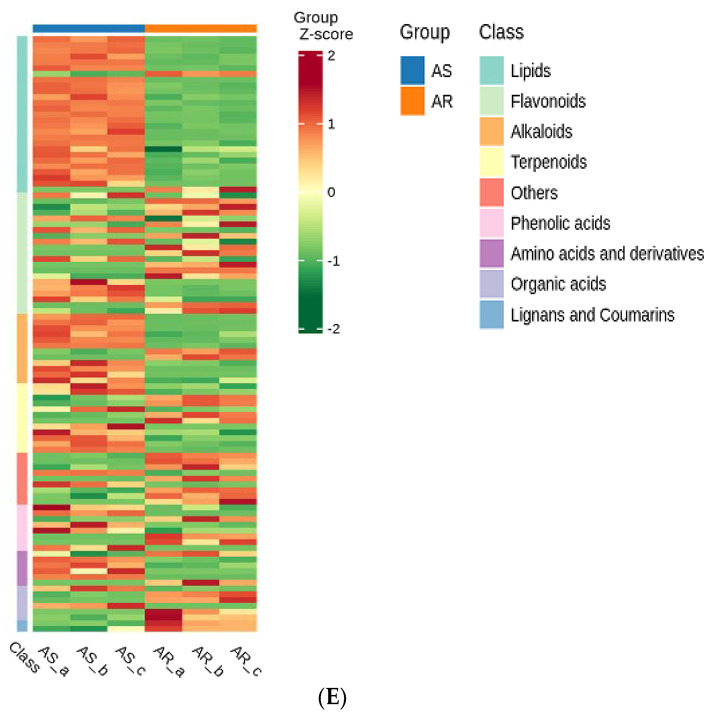
Identification of differentially accumulated metabolites (DAMs) in AR and AS pools. (**A**) OPLS-DA permutation plot for two different *Indica* rice storage pools. (**B**) Score plot generated from OPLS-DA for two different *Indica* rice storage pools. (**C**) Volcano plots depicting the expression levels of DAMs for two different *Indica* rice storage pools. (**D**) Various types of DAMs were identified in different *Indica* rice storage pools. (**E**) Overall clustering heatmap displaying DAMs for two different *Indica* rice storage pools. Each scatter represents a sample, with color and shape indicating different *Indica* rice groups, respectively.

The OPLS-DA model was utilized to identify differently accumulated metabolites (DAMs) by eliminating orthogonal variables that were irrelevant to the classification variables in the metabolites, thereby maximizing group discrimination. Pairwise comparisons between the AR and AS groups were carried out using the OPLS-DA model. The appropriateness of the OPLS-DA model was verified through 200 alignment experiments, and the pairwise comparison results showed that both the R2Y and Q2 scores exceeded 0.9 (Figure 3A).

Additionally, the well-distinguished OPLS-DA score plots manifested the differences in the metabolic profiles between the AR and AS groups. DAMs were selected based on the variable importance in projection (VIP) ≥ 1, fold changes (FC) ≥ 2 or FC ≤ 0.5, and *p*-value < 0.05, as determined by the OPLS-DA model. Volcano plots were formulated depicting the screening results (Figure 3B), revealing a total of 103 significant DAMs between the AR and AS groups, with 38 being up-regulated and 65 being down-regulated, respectively (Table S4). An overall cluster heatmap displayed all the DAMs for these pools (Figure 3C). The DAMs were categorized into nine different categories (Figure 3D), with lipids and alkaloids being the first and fourth categories of the DAMs, respectively. A total of 92.6% of lipids and 83.33% of alkaloids were down-regulated, indicating that the accumulation of lipids and alkaloids was not favorable for seed storage. As for the second category of DAMs, flavonoids were up-regulated by 57.14%, indicating that flavonoids had a considerable impact on storage resistance, and most of them were beneficial to storage resistance. Terpenoids constituted the third major group of DAMs, with 66.67% of the metabolites exhibiting down-regulation. On the whole, this observation suggested that these compounds significantly influenced storage resistance, predominantly exerting a negative impact on the seed storage resistance. Organic acids constituted the seventh group of DAMs, with 66.67% of the metabolites being up-regulated, namely, succinic acid, methylmalonic acid, isocitric acid-1-O-diglucoside, and D-malic acid. This result also indicated that most of them were beneficial to storage resistance. Interestingly, despite being the least abundant DAMs, lignans and coumarins were up-regulated, implying their potential role in terms of a positive contribution towards storage tolerance. The effects of metabolites belonging to other categories on storage resistance are more complex and require further exploration.

### 3.4. KEGG Annotation and Enrichment Analysis of DAMs in AR and AS Pools

**Figure 4:**
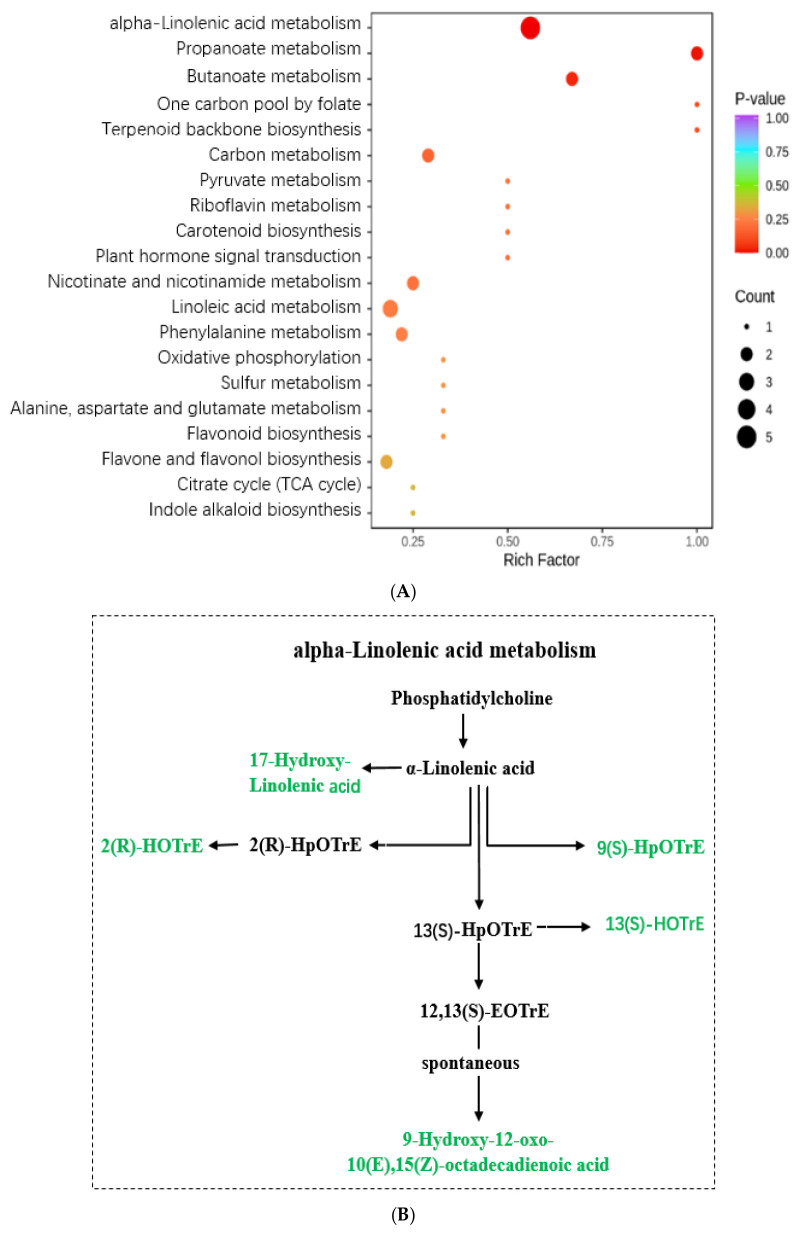

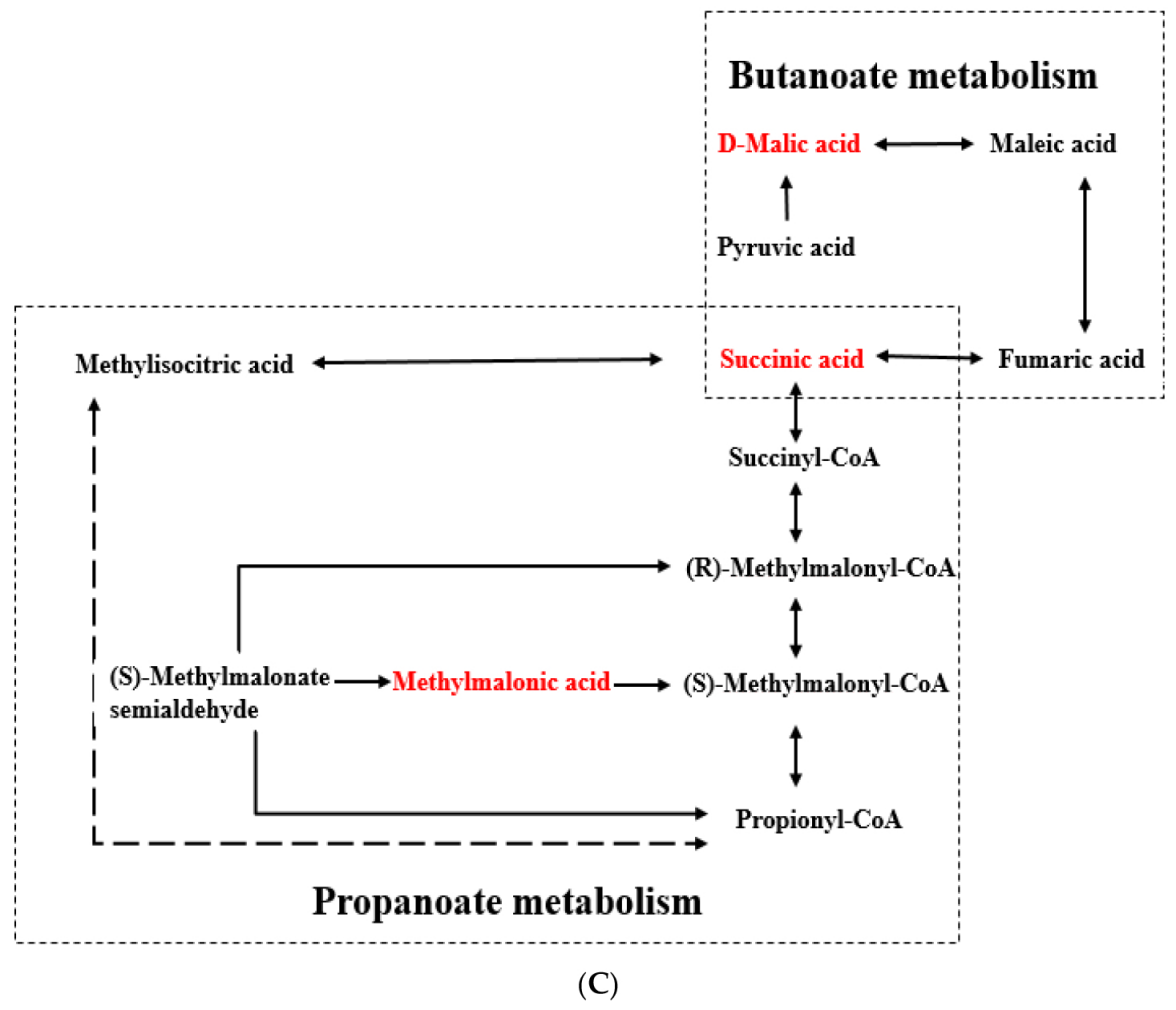
The KEGG pathway enrichment analysis of DAMs in AR and AS pools. (**A**) Bubble chart of the KEGG pathway. (**B**) Metabolite pathway of alpha-linolenic acid metabolism. (**C**) Metabolite pathway of butanoate and propanoate metabolism. The up-regulated DAMs are highlighted in red, while the down-regulated ones are indicated in green.

The KEGG database functions as a primary public pathway database, facilitating investigations into signal transduction pathways and metabolite accumulation processes [28]. In this study, we annotated and enriched the DAMs by assigning them to distinct KEGG pathways. The enriched DAMs were discovered to be associated with 38 pathways, with the major pathways illustrated through the bubble plots presented in Figure 4 and Table S5. Notably, alpha-linolenic acid metabolism, butanoate metabolism, and propanoate metabolism emerged as three significantly enriched metabolic pathways based on the statistical significance criteria set at a *p*-value < 0.05. For the alpha-linolenic acid metabolism pathway, all five metabolites, 17-hydroxy-linolenic acid, 2(R)-HOTrE, 9(S)-HpOTrE, 13(S)-HOTrE, and 9-hydroxy-12-oxo-10(E), 15(Z)-octadecadienoic acid, exhibited down-regulation. However, in both the butanoate and propanoate metabolism pathways, all three metabolites, succinic acid, D-malic acid, and methylmalonic acid, demonstrated up-regulation. The KEGG enrichment analysis revealed that the inhibition of the linolenic acid metabolic pathway could enhance seed storability, while an increased accumulation of organic acids, such as succinic acid, D-malic acid, and methylmalonic acid, in the butanoate and propanoate metabolisms was beneficial for seed storage, respectively.

## 4. Discussion

### 4.1. There Is a Significant Difference in the Metabolite Profiles Between the Two Indica Rice Pools with Different Storabilities

Seed deterioration after harvest poses a significant challenge to rice production during natural storage [29]. Previous studies have explored alterations in rice metabolites during seed storage with varying storabilities [12,30,31]. Nevertheless, limited studies have been carried out on the accumulation of initial metabolites in rice with different storage capabilities. In this study, we noticed a considerable disparity in seed storability among the *Indica* rice groups after 24 months of natural aging. We examined the differences in metabolomes between an extremely resistant storage pool (AR) and a sensitive storage pool (AS) within the *Indica* rice group. Our results disclosed 103 significantly distinct metabolites, with 38 being up-regulated and 65 being down-regulated, respectively. The main differentially abundant metabolites included lipids, flavonoids, alkaloids, and terpenoids. Furthermore, key metabolic pathways, such as alpha-linoleic acid metabolism and butanoate and propanoate metabolism, were significantly enriched in terms of the seed storability in the *Indica* rice group.

### 4.2. The Role of Lipids in Seed Storability

Lipid substances, including triacylglycerols (TAGs), diacylglycerols (DAGs), monoacylglycerols (MAGs), and free fatty acids (FFAs), are present in rice seeds. These lipid substances are susceptible to peroxidation, leading to membrane damage and the generation of toxic byproducts [32]. Lipid peroxidation products, such as malondialdehyde and acetaldehyde, are closely related to rice storability, as they can cause cell damage and intoxication through reactions with macromolecules. Linolenic acid (LNA) and linoleic acid (LA) are significant polyunsaturated fatty acids involved in peroxidation processes that contribute to cell structure breakdown, the formation of cytotoxic products, and the release of volatile decomposition products, resulting in the deterioration of rice seeds [33]. Our new findings indicated that lipids constituted the primary category of DAMs, with a significant down-regulation rate of 92.6%. This suggested that lipid accumulation was detrimental to seed storage. KEGG analysis revealed that alpha-linolenic acid metabolism was the principal metabolite pathway associated with these observations. Notably, all five identified metabolites, 17-hydroxy-linolenic acid, 2(R)-HOTrE, 9(S)-HpOTrE, 13(S)-HOTrE, and 9-hydroxy-12-oxo-10(E), 15(Z)-octadecadienoic acid, showed down-regulation within the context of alpha-linolenic acid metabolism. Consequently, it can be deduced that lipid metabolite accumulation impedes seed storage efficiency.

### 4.3. The Role of Flavonoids in Seed Storability

Flavonoids, serving as essential antioxidants for growth, development, and breeding, while enhancing stress resistance in crops [34], may function by scavenging ROS to restrict oxidative stress [35]. Proanthocyanins are a subclass of flavonoids with strong antioxidant activity, the accumulation of which could enhance the storability of rice seeds under conditions of an elevated partial pressure of oxygen (EPPO) [18]. In this study, a total of 39 different flavonoid metabolites were identified between the AR and AS groups, with 23 being up-regulated and 19 being down-regulated, respectively. Among the top twenty metabolites analyzed, five up-regulated flavonoids were identified: 5,7,5′-trimethoxy-3′,4′-methylenedioxyflavonoid; quercetin-3-o-rutinoside (rutin); Jaceosidin-7-O-(6″-O-p-coumaroyl) glucoside-4′-O-glucoside; (Z)-6-O-β-D-glucopranosyl-6,7,3′,4′-tetrahydroxyaurone; and Hispidulin-8-C-(2″-O-glucosyl) glucoside. Consequently, it can be concluded that the accumulation of flavonoids plays a crucial role in the storability of rice seeds.

### 4.4. The Role of Organic Acids in Seed Storability

Organic acids are widely utilized as antimicrobial agents within the food industry [36]. Malic acid, functioning as a food safety reagent, was disclosed to be the most effective antimicrobial acid on diverse pathogen strains [37,38]. Succinic acid was also affirmed to possess antimicrobial effects [39,40]. In this article, we ascertained that there were six differential metabolites of organic acids, with four of them being up-regulated, namely, succinic acid, methylmalonic acid, isocitric acid-1-O-diglucoside, and D-malic acid. These organic acids could potentially inhibit the activity of various microorganisms during rice seed storage, thereby enhancing the storage tolerance of rice seeds. The mechanism of the effect of organic acids on the storage tolerance of rice remains to be further explored.

### 4.5. The Role of Lignans and Coumarins in Seed Storability

Lignans consist of two phenylpropanoid units that exhibit C6-C3 linkage at the b-b’ position of the propyl side chain, showing stereo-selective oxidative coupling and the different substituted patterns of aromatic moieties [41]. Lariciresinol, belonging to lignans, is an antioxidant [42] and antiviral [43,44] compound. Coumarin is a fragrant organic chemical compound of the benzopyrone chemical class that is a natural substance found in many plant species, which exhibits a variety of potent pharmacological activities, including antioxidant, antibacterial, anti-inflammatory, antitumor, and antiviral activities [30]. Eleutheroside B1, a coumarin compound, has a wide spectrum of anti-human influenza virus efficacy [45]. In this study, we found that Lariciresinol and Eleutheroside B1 were up-regulated in the aging-resistant *Indica* pool. Thus, both of these substances could potentially inhibit the activity of various microorganisms during rice seed storage by means of antioxidant, antibacterial, and antiviral activities, thereby enhancing the storage tolerance of rice seeds.

## 5. Conclusions

We identified the storability of global rice core germplasms stored under natural aging conditions for two years. Subsequently, we conducted a comprehensive widely targeted metabolite analysis of UPLC-MS/MS on two different *Indica* rice pools with significant seed storability differences and identified 103 DAMs between them. Further analysis found that the aging-resistant rice pool demonstrated higher accumulation levels of flavonoids, terpenoids, phenolic acids, organic acids, lignans, and coumarins while exhibiting a lower lipid content and alkaloid levels compared to the aging-sensitive rice pools in mature seeds. KEGG pathway enrichment analysis indicated that the inhibition of the linolenic acid metabolic pathway and an increased accumulation of organic acids, such as succinic acid, D-malic acid, and methylmalonic acid within butanoate and propanoate metabolism, were identified as a beneficial factor for seed storage under natural aging conditions.

## Figures and Tables

**Table 1:** The storage performance of selected *Indica* rice pools in this study.

No.	Name	Pool	K9 Group	Mean Germination Rate During Natural Storage (%)	
				3 Months	24 Months
C068	IRIS_313-11607	AR	XI-2	96.67	97.33
C334	IRIS_313-9966	AR	XI-1B	82.67	98.67
C337	IRIS_313-10109	AR	XI-3	95.33	94.00
C354	IRIS_313-10333	AR	XI-1B	100.00	99.33
C418	CX226	AR	XI-1B	100.00	96.00
C100	IRIS_313-11945	AS	XI-2	90.00	6.67
C227	IRIS_313-10681	AS	XI-3	94.67	0.67
C228	IRIS_313-10928	AR	XI-3	96.67	0.00
C244	IRIS_313-11804	AS	XI-1A	91.33	4.67
C287	B094	AS	XI-1A	98.67	0.00

## Data Availability

The original contributions presented in this study are included in the article. Further inquiries can be directed to the corresponding authors.

## Supplementary Materials

The following supporting information can be downloaded at: https://www.mdpi.com/article/10.3390/metabo15010019/s1, Table S1: Basic information of 375 rice accessions; Table S2: The average germination rate in the whole population after storage for 3 and 24 months under natural aging conditions; Table S3: Complete list of the measured metabolites in AS vs. AR groups; Table S4: Different accumulated metabolites in AS vs. AR groups; Table S5: Metabolic pathways of differential metabolites in AS vs. AR groups.
